# What Do ‘Humans’ Need? Sufficiency and Pluralism

**DOI:** 10.1080/21550085.2023.2247814

**Published:** 2023-08-29

**Authors:** Ben Davies

**Affiliations:** Uehiro Centre for Practical Ethics, University of Oxford, Oxford, UK

**Keywords:** Sufficiency, cognitive disability, pluralism, need

## Abstract

Sufficientarians face a problem of arbitrariness: why place a sufficiency threshold at any particular point? One response is to seek universal goods to justify a threshold. However, this faces difficulties (despite sincere efforts) by either being too low, or failing to accommodate individuals with significant cognitive disabilities. Some sufficientarians have appealed to individuals’ subjective evaluations of their lives. I build on this idea, considering another individualized threshold: ‘tolerability’. I respond to some traditional challenges to individualistic approaches to justice: ‘expensive’ tastes, and adaptive preferences. Finally, I end by offering some suggestions about how this relates to policymaking.

## 1. Introduction

The idea of sufficiency is common to both environmental discussions and philosophical theories of justice. In both cases, certain thresholds are established. Justice sufficientarians are interested in the point at which the strength of our claims on each other stops, weakens, or otherwise changes. Ecological sufficientarians are interested in the maintenance of our environment to sustain a particular standard of living for humans, along with other goods such as the natural world. Thus, both views will distinguish between standards of living which people are entitled to, and standards which might be nice to have but which are not entitlements, and perhaps must be sacrificed to ensure that everyone can have enough.

In this paper, I largely take this schematic, general approach to sufficiency that is common to both fields. There are of course important differences between the ways of understanding sufficiency in each domain. But I am interested in a common assumption made in ecological and justice sufficientarian discussions: that the goods which can be given this special status of entitlement must be those which are universally valuable to humans. Thus, while I illustrate my argument with some examples of environmental concern, my aim is not to engage in detailed consideration of environmental justice but rather to raise some theoretical background issues that are common to both distributive and environmental discussions.

Section 2 outlines a commonly raised, general problem for the idea of sufficiency: that any sufficiency threshold will be morally arbitrary. Section 3 describes the most common response to this problem, which is to introduce universal goods as the basis of a sufficiency threshold. Section 4 raises some challenges for this approach: any more than the most minimal goods required for survival are not truly universal. Section 5 offers another approach, which could be adopted instead of or as well as putatively universal standards: a focus on individual experience, and in particular individuals’ contentment with and toleration of their situation. Section 6 considers a further challenge to this move: the problem of hedonic adaptation. Section 7 briefly considers application in policy.

## 2. Sufficiency and Arbitrariness

Sufficientarians assign importance to at least one threshold when deciding how resources ought to be (re)distributed. The idea of sufficiency is also relevant to environmental and ecological concerns. Humanity must reduce our carbon emissions, pollution, and other environmental impacts. Transitioning to a more sustainable way of living will have costs, at least in the short and medium term. The idea of sufficiency might play two roles in this context. First, it can mark certain goods that justify taking on these costs: if we fail to act, many people will—unjustly—be forced into insufficiently good lives. Second, sufficiency may help us to decide how to allocate costs: all else being equal costs should fall on those who are above the sufficiency line, and should not threaten anything which is essential to them having a sufficiently good life. As Shue (2014, p. 64) notes, it surely makes a difference that some emissions people produce are used in the context of feeding their children, whereas others are used for luxury cars.

Different sufficientarians assign different roles to sufficiency thresholds, and locate it at different points (Crisp, 2003; Frankfurt, 1987; Haybron, 2020; Huseby, 2010; Meyer & Roser, 2009; Nielsen, 2018). But no matter what our answer to these questions, the idea of sufficiency faces a central, difficult challenge: fixing thresholds non-arbitrarily. Thresholds entail moral *discontinuity* (Benbaji, 2006; Chung, 2017): we should treat two people differently if one is above and the other is below the threshold. Which side of the threshold you end up on might turn on small differences. If the threshold is arbitrary, two people separated by only a small difference will arbitrarily be treated differently.

Sufficientarians have two options. The first is to explain why arbitrariness is sometimes acceptable. For instance, we might have to draw a line somewhere, but any particular place we draw it may be somewhat arbitrary. The second option is to find a non-arbitrary threshold, where although moving across it may involve only a small change, that change is normatively significant (Benbaji, 2005, p. 323). Many sufficiency theorists pursuing this option have tried to outline *universal* thresholds. I suggest that this causes difficulty when considering the pluralism of human lives, including individuals with significant cognitive disabilities, and explore the prospects for an approach which includes consideration of subjective attitudes. I argue that subjective attitudes are apt for avoiding the threat of arbitrariness, invoking two morally significant points which people may reach from a subjective position: being content with their situation, and finding their situation intolerable. These points are such that one can arrive at them through only a small change in one’s circumstances, but reaching either of then makes a significant difference to the moral status of one’s situation.

## 3. Universal Thresholds

The usual place for sufficientarians to turn in seeking non-arbitrariness is putatively universal human goods. In healthcare, for instance, Ram-Tiktin (2012, 2016) emphasizes ‘basic human functional capabilities’ based on systems of physical and psychological capabilities needed to execute one’s ‘life plans’. While the idea of life plans is individualized to some extent, indexing it to the idea of biological functioning aims to involve some level of universality. Shields (2016, pp. 44–81) emphasizes the importance of having the capacities required for autonomy, as does Nielsen (2016). Autonomy is necessary for ‘most’ people in Hassoun’s (2021a) account of the ‘minimally good life’ (p. 334). Axelsen and Nielsen (2015) define sufficiency as ‘freedom from duress’, which requires not facing significant pressure against or obstacles to success in ‘central areas of human life’, i.e. ‘aspects of life that humans have in common’ (p. 409). And Benbaji (2006) sets one of several sufficiency thresholds at the point of possessing ‘person-making capacities’, explicitly including rationality and the ability to form complex desires (pp. 339–40).

We also see frequent appeals to universality in theories of ‘basic needs’, which have been a focal point both in health ethics and in thinking about sufficiency in relation to consumption and ecological crisis. Many proponents of basic needs appeal to universality to ground their moral force (Benbaji, 2005; Clark Miller, 2012; de Campos 2012; Doyal & Gough, 1991; Gough, 2017, 2020; Meyer & Roser, 2009; Meyer & Steltzer, 2018; Page, 2007; Rawls, 2001, pp. 47–8; Reader, 2007; Reader & Brock, 2004; Schramme, 2019; Shue, 1981; Wiggins, 1998). These needs operate at different levels, including requirements for subsistence, such as certain calorific intake and access to oxygen, and higher-level requirements, such as those involved in avoidance of significant harm, which may include social participation as a necessity to further our goals. One common way of marking this distinction is to distinguish between ‘subsistence’ and ‘luxury’: the things we need are universal subsistence goods; the things we merely want are luxuries, contingent on particular, individual desires. There is nothing wrong with luxury, unless having it comes at the cost of others’ subsistence.

In both areas, we thus find attempts to ground sufficiency on *general purpose requirements* for the advancement of more specific goals. As such, these general-purpose requirements take on a distinctive moral weight compared with other goods.

Appeals to universal thresholds seem attractive as solutions to the arbitrariness problem due to two features. First, they are plausible as *non-reducible* demands of justice. This matters because if we are only interested in a particular good because it is instrumentally valuable for some further good, which we do not think should be distributed in a sufficientarian way, then our fundamental theory of justice is not really sufficientarian. Autonomy is certainly instrumentally valuable. But it is also intrinsically valuable; we care about controlling our own lives for its own sake, not just because it produces better results. Second, and more centrally to the arbitrariness worry, these really do seem to be cases where a small difference on some metric (e.g. in capacity, in calorific intake, in the obstacles one faces) makes a *big* difference to one’s entitlements, i.e. to the moral significance of one’s situation. The difference between facing obstacles that are just manageable and ones which are just overwhelming may be descriptively small, but is normatively significant. So is the difference between having minimal autonomy and just falling short.

Thus, appeals to these universal standards have formed an important bulwark against charges of arbitrariness. As I will now suggest, however, such appeals are not without problems.

## 4. Pluralism and Disability

The claim of universality—that the identified threshold applies to all subjects of justice—is most plausible regarding extremely low thresholds; consider again that we all need oxygen to survive. Generalising, the threshold of ‘bare subsistence’ is universal. But bare subsistence is not a plausible threshold for sufficiency, since it groups everyone whose bare subsistence is not at risk together. Even given the fundamental importance of subsistence, the idea that all existence above this should be seen as ‘luxury’ is not plausible.

One option is to try to ground the theory in a higher but still universal threshold of a particular standard of life, such as a ‘good human life’ (Ram-Tiktin, 2016); ‘central areas of human life’ (Axelsen & Nielsen, 2015); a ‘minimally decent’ life (Miller, 2007, p. 181; Shue, 1993, p. 42); or a ‘minimally good life’ (Hassoun, 2021a, 2021b). Common to these ideas is the thought that we can accommodate diversity in the values that people hold; we do not presume a one-size-fits-all view of what a valuable life involves, but focus instead on the common requirements for pursuing a range of values, especially through appeals to autonomy or freedom. Nonetheless, the idea of a minimally good life retains universality because humans share certain needs, including the need to be able to plan their own lives.

Such appeals are significantly more demanding than bare subsistence, and thus more plausible as a point where claims of justice run out or weaken. A further restriction imposed by the idea of ecological sufficiency might be that the life plan I pursue with these general-purpose means must be compatible with the plans that others wish to pursue.

However, the apparent universality of such ideals is called into question when we think about some groups of individuals whose interests are often neglected in discussions of both distributive and environmental justice. I will primarily focus on this consideration as it arises concerning individuals whose cognitive disabilities render them either fully unable to develop goals or significantly reduce their capacity to develop and pursue long-term plans. As Smith (2013b) puts it, ‘the capabilities to plan for the future and make meaningful choices can be seriously compromised with people who have diminished cognitive abilities’ (p. 28).^1^ The pursuit of autonomy, negative liberty, or independence may look misplaced for individuals who lack such capabilities (see also Raz, 1988, p. 299). But it may also arise in other contexts. For instance, the idea of life plans in any detailed sense seems unavailable to most non-human animals; yet from the perspective of ecological sufficiency, animals’ interests are of considerable importance. Similarly, even if young children *will* grow into adults who can form life-plans, and therefore have future-directed interests on that basis (such as an interest in education), their present interests are not exhausted by such considerations.

I want to take care here not to over-generalize. The suggestion is not at all that a person with just any cognitive disability is incapable of forming goals or life plans; that is clearly untrue for many people, and for them autonomy, liberty and independence are deeply important (Brown et al., 2019; Palynchuk, 2022; Schmitz, 2013; Shakespeare, 2014, p. 61; Silvers, 1995; Smith, 2013a), though may require particular structures to achieve (Reynolds, 2022). But there *are* individuals whose formation of goals is extremely attenuated or absent (Campbell et al., 2021, p. 711).

My claim is that individuals who are constitutively incapable of autonomy and significant independence, and for whom (certain kinds of) negative freedom would not be beneficial, can have good lives. Moreover, these capacities (or the lack thereof) are not relevant to their having good lives (though see Gould, 2022). Their form of flourishing (*pace* Nussbaum, 2006) is not a shadow of our own but is, rather, different in important ways. This does not mean that the good life of someone with significant cognitive disabilities is entirely alien to us, but simply that something which has been taken as central by many people to the flourishing of the lives of people *without* such disabilities may not apply.

Again, I want to stress that I am not making this claim about all people with cognitive disabilities, even significant ones. I also restrict my claim in the other direction; I do not claim that there are *no* forms of disability that prevent people from having good lives. Finally, it is important to emphasize that such a pluralist outlook does not (intentionally) exclude individuals with certain cognitive disabilities from ‘the human’—either conceptually or in terms of moral status—though I acknowledge that in practice this interpretation is a significant risk (Riddle, 2013). Rather, it is a denial of the claim that a good human life must contain capacities such as autonomy, or involve the kind of independence others take for granted as essential (Carlson, 2015; Garland-Thomson, 2012; Schmitz, 2013, pp. 56, 59). In other words, it is an (attempted) expansion of what it means to be human, not a restriction.

However, this does not mean that we must abandon or deny the centrality of autonomy to the lives of those who *can* exercise it (Wasserman & Asch, 2013, p. 151). Precisely what I reject is the idea that if a requirement is not universal, then it is not sufficiently basic to ground a sufficientarian view (see also Brison, 2021). The capacity to form and pursue a life plan has significant value for me, and likely for you as well. If that capacity is severely restricted or frustrated, then it will be difficult for me to live well. This has to do with the kind of creature that I am, but the relevant kind here is not ‘human’. Rather, the relevant kinds are twofold. The first is simply being a creature with certain capacities. Having those capacities is a pre-requisite to valuing them; this is not true of all capacities (I can wish I could play the violin well without being able to) but it is true of certain cognitive capacities (Yelle, 2016). The second relevant kind is *the particular individual that I am* (Moller, 2011, p. 202) Autonomy matters to me not because only an autonomous life can be good, but because imagining myself without autonomy is not to imagine *myself* at all.

So, I suggest that we should accept that the central liberal idea of autonomy to which many sufficientarians appeal constitutes a plausible threshold for many subjects of justice, but not all. This then raises two questions about appealing in our theory of justice to autonomy as a universal ground for the good life.

The first concerns what our theories of justice express about individuals who cannot achieve what we have defined as a good (enough) life. A theory of justice which sets a single, universal standard for a good life tells us that those who fail to meet that standard cannot live well. Setting and pursuing a vision of the (minimally) good life is unachievable for some individuals with significant cognitive disabilities. And yet while some such individuals do in fact have bad lives, this is not because they are *incapable* of living well. Such a judgment has potentially significant practical implications, for instance in the provision of healthcare when not all patients can be treated, or in arguments about whether an individual’s quality of life makes providing *any* life-sustaining care in their best interests (Hellman & Nicholson, 2021; Miller Tate, 2022; Scully, 2020; Wilkinson, 2021). Moreover, this implied judgment goes against the informed opinion of many people who care for individuals with significant cognitive disabilities. Such individuals *can* have good lives, for instance because they are happy, or are members of loving relationships (Campbell et al., 2021; Kittay, 2009; Shea, 2019). A theory of justice which implies that they cannot gets things importantly wrong.

We also need to consider the position of individuals who are constitutively incapable of certain central capacities within our theory. In other words, how does our sufficientarian theory tell us to behave toward those who will not, no matter what we do, achieve ‘sufficiency’ as the theory defines it?

One potential response is that although some individuals will never achieve sufficiency (understood as essentially involving autonomy), there is still value to getting them *closer to* the threshold, and that such improvements have greater moral weight than equivalent improvements for individuals above the threshold (Hirose, 2016; Nussbaum, 2006, 2011; Zameska, 2020).

Thus we have the view that:

1. there is a universal, objective sufficiency threshold, namely that concerning what is commonly needed to pursue a good (enough) life;
2. some people cannot achieve that threshold, no matter what we do; and
3. we should not abandon the individuals covered by (2), but instead move them closer to the threshold identified in (1).

Such a view is clearly well-intentioned. But the original rationale for focusing on a universal threshold was to avoid the charge of arbitrariness. Goods such as autonomy do this because a small change can make a significant moral difference. The *achievement* of a level of autonomy sufficient to form and pursue life plans may be descriptively close to just barely lacking that capacity; and yet the change in value may be enormous. But it is less clear that this reasoning explains why we should want people to be closer rather than further from our threshold if there is no chance of them ever clearing it.

The key challenge here is in explaining why getting closer to this threshold is valuable for the individual. If we think about thresholds cashed out in negative terms (such as freedom from duress), we may find the opposite problem: *achievement* of the threshold does not improve, and may worsen, some individuals’ lives. An individual who is highly dependent might strictly meet the criterion of facing no significant pressure or obstacles to achieving a good life but nonetheless fail to live well at all if they are not provided with sufficient support. Importantly, while their life can clearly be improved, it might not be improved by moving them further along the dimension by which sufficiency has been defined.

My suggestion is that these problems should prompt sufficientarians to think more about subjective quality of life in a more individualized sense. While I have raised this issue centrally for individuals who cannot hope to achieve autonomy, the discussion in the next section may also prompt us to think about subjectivity for those of us for whom autonomy and freedom are of significant value.

## 5. Contentment and Tolerability

The central claim of this section is that justice is not merely concerned with what one can ‘do and be’ (Sen, 1979), or with the effect of various goods on opportunity (Daniels, 2008). It is also concerned with how people’s lives seem and feel to them from the inside. I consider two such bases for sufficiency that respond to this concern, one of which (contentment) has been advocated by other sufficiency theorists, and one of which (tolerability) has not, as far as I know. I then explain how these considerations might avoid the problem of arbitrariness.

### 5.1. Contentment

The idea of a sufficiency threshold which refers centrally to a person’s evaluation of their own situation is not new. The central concept appealed to by some sufficientarians is the idea of contentment (Frankfurt, 1987; Huseby, 2010, 2020).^2^

For Frankfurt, contentment is consistent with thinking that more would be better, but it precludes an ‘active interest’ in having more. For instance, consider an example with relevance to the environment: diet. Someone might be content with a vegetarian diet, which in general produces lower carbon emissions than an omnivorous one (Ritchie, 2020), simply because that is what they were brought up eating, or it is what most people eat around them. But they might know that if someone happened to give them meat they would enjoy it, perhaps more than their current diet. Frankfurt (1987) argues that just as you might be satisfied with your current diet, so too might someone be satisfied ‘with the amount of satisfaction he already has’ (p. 40). Thus, contentment can have a self-referring quality; one might recognize that one could be happier but be content with one’s current mood.

There are at least two ways to understand contentment in Frankfurt’s sense. One is as a form of pure satisficing (Byron, 2004). Whereas many theorists have assumed that, from a purely self-interested view, rationality demands maximizing or optimizing the goods of life, pure satisficing involves settling for a particular level of goods that one finds satisfactory. Thus on Frankfurt’s view, to be ‘content’ is to recognize that one could do better, but to not be motivated to seek to do better. What makes this a pure form of satisficing is that the reason for not seeking to do better is *not* the fear that this will end up costing more than it is worth, e.g. in time or money. This would be merely instrumental satisficing, i.e. settling for a satisfactory level in one respect because that is the optimal strategy overall. Frankfurt’s account does not involve any such optimizing calculation: a person who is content simply does not seek to improve their situation. Many people regard such pure satisficing as irrational.

The second interpretation of contentment is in terms of instrumental satisficing. One might recognize that there is a sense in which one could do better, but believe that there is too much risk or cost in aiming for this. This is a more difficult ground for a sufficiency threshold, since at least in some cases society, the state or some other individuals could make it *less risky* for you to try to achieve more. In such cases, the appeal to ‘contentment’ seems potentially question-begging, since we might ask whether you should in fact be in this situation.

Huseby’s appeal to contentment is in defining a good life; as we have seen, this is an important but under-examined feature in many sufficientarian theories. Huseby thinks contentment is consistent with desiring more, but being ‘satisfied’ with one’s overall quality of life. His important addition to Frankfurt’s view is that not just any contentment will do. For Huseby, one must be content *for good reason*. For instance, one should not be content because one has been manipulated.

On both accounts, contentment involves the possession or absence of an attitude about one’s overall situation. As such, it also faces a problem when applied to some individuals with significant cognitive disabilities. If contentment requires a positive attitude involving detached consideration of one’s overall situation, then some individuals with significant cognitive disabilities will never be ‘content’, since they cannot take such a detached perspective; thus, we again get the result that such individuals cannot achieve sufficiency. If contentment is the absence of a desire to have more, then such individuals may count as content even if they are subjectively quite unhappy, by virtue of an inability to form the relevant sort of desire.

As with goods such as autonomy, I do not take the non-universality of such attitudes to render them irrelevant for questions of justice. For many of us, *one form of contentment* does involve the taking of a somewhat detached perspective on our lives. The fact that such a perspective is not universally available does not render it irrelevant to sufficiency. Rather, it means that we need to think more carefully about how contentment applies to individuals who lack this perspective.

While acknowledging the importance of the intellectualized, detached notion of contentment, it is also important to focus on its felt qualities. For as well as the idea of contentment that Frankfurt and Huseby employ, there is also a more immediate idea of a particular feeling. I hope that all readers are aware of that feeling, though some will experience it more than others; it is a relaxed, gentle, pleasant feeling that arises from having no immediate worries (or, at least, being able to put them to one side for the moment) and being immersed in a pleasurable moment. Such a feeling does not require any detachment from one’s situation; indeed, those of us who are too detached may find it harder to achieve. But it is another sense of contentment that is important.

As Huseby says, that a person enjoys such subjective, short-term feelings is not sufficient to think that they have what justice requires. Feeling this way can come about via manipulation, or failure to understand how bad one’s situation is. Yet for all that, such moments are important; to be able to access them, and not infrequently, is a *reliable* if not infallible marker of having much that one needs and wants, and of being in a sufficiently stable situation as to be able to suspend worries for the time being. Such subjective contentment is compatible with a wide range of different sets of needs, and is one common way of having a decent life. A person who is content in this subjective sense needs nothing more, at least right now. And a person who is regularly content in this way likely has a good life on their own terms.

The central challenge which an appeal to goods such as autonomy was meant to avoid was the problem of arbitrariness. Does the idea of contentment reintroduce this worry? I think not. The arbitrariness challenge is to find a threshold where individuals on either side of it are descriptively similar, but where we have some moral grounds for treating them quite differently. And the fact that someone is *content* with her life is, I suggest, a morally significant achievement. The moral difference it makes may not be absolute: it is not that we never have reason to benefit the contented, nor that we always have reason to benefit the discontented. But along one dimension – subjective satisfaction – contentment may provide a non-arbitrary basis for distinguishing between people.

### 5.2. Tolerability

So, some sufficientarians have turned to subjectivity to sketch out a threshold. However, sufficiency thresholds typically serve two purposes. These are (i) marking the point above which claims of justice weaken or end entirely; and (ii) marking a point where people’s claims to benefits are particularly morally urgent. The idea of contentment may serve this first role, but it is not plausible to serve the second role: this would imply that everyone who is not content is of urgent moral concern.

Indeed, it is doubtful that a single threshold can serve both roles. As such, some sufficientarians adopt a multi-threshold view, where an upper threshold serves the first role, and a lower threshold serves the second. It is interesting that while Huseby and Frankfurt seem to take a subjective approach to an upper threshold, the same cannot be said for the idea of a lower threshold. Frankfurt does not explicitly write in these terms, but Huseby’s multi-threshold view uses subsistence (2010) and the idea of basic needs (2020) as the grounds for a lower threshold. In this subsection, I suggest that the lower threshold should also be concerned with how our lives seem to us from the inside, in the form of tolerability.^3^

While the idea of tolerability does not—so far as I am aware—appear in the sufficientarian literature, it does make an appearance in some writing on healthcare. For instance, the Nuffield Council on Bioethics (2006), considering the care of critically ill infants, suggests that a key question is whether further treatment would place an intolerable burden on the patient (the report also uses the cognate term ‘unbearable’) (also Coggon, 2008; Kopelman, 1997). And the idea of unbearable suffering has been central in debates around physician-assisted suicide, where Dees et al. (2010) note that it ‘has not yet been defined adequately’ (p. 339). Similarly, we might think that a significant injustice of the climate crisis is that it forces those who are most vulnerable to live in intolerable circumstances, while those of us who are wealthy have much more than we need.

Like the idea of contentment, the idea of tolerability can be understood in a more or less intellectualized way, though the distinction is based on somewhat different grounds. What we find tolerable is often affected by how we understand a situation. For instance, a particular level of pain might be intolerable in one context but made tolerable by knowing that it will end in 30 seconds, or is necessary to help someone I love. In a more intellectualized sense, toleration is a reflective attitude we can take toward our lives.

For many of us, including individuals with cognitive disabilities, tolerability can be seen through several sorts of evidence. Pain is perhaps the most straightforward example. Stopping enjoyed activities suggests that pain is not compensated by (certain levels of) pleasure. Where the foregone gains are significant, this suggests that the pain is intolerable when undertaking valued activities. Similarly, accepting other costs (such as sluggishness from strong painkillers) is a sign that the pain is significant enough for its relief to be worth those costs. Where the costs are significant, this suggests that the pain is intolerable. Intolerability can also be seen through significant emotional distress or shutdown.

The above discussion does risk glossing over an important question, which is whether the idea of something being intolerable should be taken literally. It depends on exactly what we mean by ‘tolerate’. People carry on with their lives under sometimes extraordinary levels of suffering; should we say that their suffering is tolerable because they are able to ‘carry on’? If so, then tolerability will not serve as a useful lower threshold. I want to suggest, though, that we can have a slightly less strict understanding of this idea, where a person *literally* bears their burdens, but finds them to be crushing in one or more of a variety of ways: grinding them down emotionally, deadening their enthusiasm for life, making them question whether life is worth it, causing significant distress, or rendering them unable or unwilling to pursue previously valued opportunities (Dees et al., 2010).

I suggested both behavioral and emotional evidence for intolerability. Only these latter examples seem relevant for individuals with the most significant cognitive disabilities, those who cannot make trade-off decisions. And only some notions of intolerability in the previous paragraph apply. On the other hand, with respect to pain the bar for what is tolerable may be much lower, at least insofar as individuals with some kinds of disabilities are unable or less able to contextualize their suffering in ways that make it bearable.

The idea of tolerability seems to me extremely important when we think about the bare minimum that we owe people as a matter of justice. To reiterate, the suggestion is not that so long as people can tolerate their lives, they have all they are entitled to. Rather, the idea of tolerability marks a point where it is of distinctive moral importance that we benefit people, making their lives *at least* tolerable for them. Again, this is relevant to the question of arbitrariness. The difference between a person who finds their situation intolerable and one who can just barely tolerate it may be slight. Both have strong claims to their situations being improved. But the person who finds their situation intolerable has a significantly stronger claim at least in this respect.

In the next section, I consider a central objection to including such assessments like contentment and tolerability in determining justice. Before doing so, however, it is worth noting that including consideration of how people’s lives seem to them does not mean that this is the only metric of justice. It is entirely consistent with everything argued thus far to say that certain kinds of reasons for discontent, or even for finding one’s life intolerable, are personal matters and not relevant to justice. For instance, if we think that principles of justice should regulate only public institutions and practices, a person’s discontent because of some particular issue in their romantic life may not be relevant to justice. Alternatively, as I outline in more detail in the next section, certain kinds of values that lead to discontent—such as the racist who is discontented with political equality—can be legitimately ignored. And we may well accept Huseby’s suggestion that certain origins of contentment or toleration, such as manipulation, are disqualifying. Thus, we need not say that it is the business of the state to *ensure* that people are content, or that their lives are tolerable. Rather, the notion of contentment provides us with a point beyond which it is less important to benefit people in ways that are within the state’s purview. And the idea of tolerability might be restricted to certain areas which are state responsibilities. It is no strong objection to caring about people’s subjective evaluations of their lives that people can be unhappy for all sorts of reasons that are no business of government (Bickenbach, 2013).

## 6. Expensive Tastes and Adaptive Preferences

This section considers and responds to some familiar challenges to views of justice which rely significantly on individuals subjective assessments of their own lives: ‘expensive’ tastes, and adaptive preferences.

The problem of expensive tastes concerns individuals who are unsatisfied unless they have a quality of life far better than that which most people would accept. Though in its original formulation this is a non-comparative preference for resources which are literally expensive (Dworkin, 1981), we might also imagine an individual with *comparatively* expensive preferences, e.g. to be the richest person in the world. Essentially, it is unfair to give the same weight to making such individuals content as we do to those with more ordinary tastes *or* to those with different kinds of expensive tastes, namely those for whom the fulfillment of ordinary preferences costs more (this latter group may include some individuals whose disabilities mean they require equipment for everyday living).

The problem of adaptive preferences is the idea that people whose lives involve significant burdens or deficits become inured to them, and thus express contentment with lives that others would not accept. Whether this applies to disability itself is contentious. But it is at least a broader issue for any theory of justice which relies in part on subjective attitudes.

Both problems are concerned with hedonic adaptation (Bickenbach, 2013), which involves individuals returning to roughly similar levels of subjective happiness or life satisfaction even after significant gains or losses. We need not accept a strong version of hedonic adaptation either as universal or as the idea that individuals have a single, unchanging happiness default (Haybron, 2020). Rather, the idea that people will often adjust to new circumstances, changing their goals to reflect either acquired challenges or new capacities and resources, means that a focus on people’s subjective assessments of their own lives will likely tolerate a greater level of inequality than would a focus on objective goods. A key worry here is that since we are concerned with an actual world which involves considerable levels of inequality, caring about contentment and tolerability will—given people’s adaptation to their current lifestyles—conservatively reinforce the status quo. However, proponents of subjective attitudes’ relevance can make several responses.

Take first the issue of expensive tastes. I have said that expensive tastes can come in two kinds: the traditional variety, which are non-comparatively expensive; and a comparative variety, which are preferences to maintain a certain position relative to others. The latter kind are easier to deal with, since they are essentially a form of what Cohen terms ‘offensive tastes’ (1989, p. 912). This is something of a misnomer, since the problem with offensive tastes is not that they are offensive, but that they are predicated on others’ suffering or oppression. For instance, if a person finds it intolerable to live and work with people of other religions, they have no claim on the state or others to do anything about what they find intolerable. Positional preferences are another example: to find it intolerable not to be rich enough to wield great power over others is not a preference that anyone else has reason to satisfy (Lippert-Rasmussen, 2013).

Non-comparative expensive tastes are more difficult. For instance, consider the preference some have for taking three or four holidays abroad by plane each year, or having the heating running at 30°C. Such individuals might claim to find life without their holidays, or with the thermostat turned down, ‘intolerable’. Importantly, there is nothing intrinsically wrong with such expensive preferences. Yet if we use the idea of tolerability as a marker of importance, we seem to be bound to treat these wealthy individuals’ claims of intolerability as equivalent to those whose lives are made intolerable through severe hunger, extreme weather, or displacement. If this is an implication of my argument, then my argument is implausible.

We can make three partial responses here. The first is that insofar as achieving tolerability for someone is expensive, doing so will often come at the cost of achieving tolerability for multiple others. Where there is a trade-off between achieving tolerability for more or for fewer people, there is a defeasible case for doing the former.

However, this response is incomplete because it treats *all* cases where tolerability is resource-intensive on a par. The individual whose life is intolerable because of physical illness or material deprivation that they can neither control nor relieve clearly has a stronger case than the individual who finds their situation intolerable despite having a life that most would enjoy. Thus, a further priority ordering is important: the more difficult and more costly it would be for an individual to alter their preferences, making a currently intolerable situation tolerable, the greater their claim.

Finally, it is worth noting that when we deal with the idea of sufficiency at the political level (rather than at the level of bare moral value), we cannot help but use heuristics and generalizations. I address this in further detail in the final section, where I discuss the application of this theory to policy. However, briefly, a person’s claim to find their situation intolerable may be misleading: they may be exaggerating, lying, or simply not really thinking about how much worse life could be. Thus, while claims of intolerability for those who appear to have everything they need should not be dismissed out of hand, we can set a higher evidential bar for such claims than for those from individuals who lack more common requirements for a good life.

Turn now to adaptive preferences. First, it is worth restating that I have not endorsed the claim that it is *only* our subjective self-evaluations which matter. Thus, the position outlined here does not say, for instance, that if someone is content then they have no entitlement to more. Second, as well as having multiple thresholds in a vertical sense (i.e. upper and lower thresholds), sufficientarians can be horizontal pluralists, whereby there are different aspects of sufficiency which are not fully fungible (Pedersen & Nielsen, in press). On this view, the fact that someone is content with what they have marks one important form of sufficiency, but not the only one. It also means that claims to further benefit either cannot be made (on a strong view of the role of the upper threshold) or have a weaker force *on the basis of their effect on a person’s subjective states*. But it could be made on some other basis.

Relatedly, I repeat that the idea of tolerability also does not set a point at which individuals have no further claims of justice; decoupling that claim from the positive claim that a lower threshold marks a point of significant urgency was precisely the purpose of a multi-threshold approach. Thus, it is no implication of my view that those who adapt their preferences so that they find very bad circumstances tolerable are entitled to nothing more. Rather, the claim is that a person’s *finding their situation intolerable* justifies a special moral concern with helping them. Even if we think that some people make a mistake in tolerating, or even feeling contentment at, situations that are objectively bad, it is still worse in one respect to live in a bad situation *and find it intolerable* (Jølstad, Forthcoming). Thus, while someone’s finding their situation tolerable does not remove our reasons to help them, someone in a similar position who finds it intolerable is thereby worse off, and thus has a stronger claim to benefit.

Third, a sufficientarian theory of distributive justice is not a complete theory of political rights and obligations. It may be important or even obligatory for societies to avoid certain kinds of social or economic relations for independent reasons (Fourie, 2021).

Finally, it is important not to exaggerate the scope of the problem. As many authors note (Albrecht & Devlieger, 1999; Barnes, 2016; Smith, 2013a), people who acquire significant disabilities may adapt to them through critical reflection and engagement with their new capacities and social identities, thereby developing new preferences. Though such preferences are ‘adaptive’, there is nothing wrong with them; indeed, it would be a sign that something had gone seriously wrong if people never adapted our preferences to changes in our circumstances.

## 7. Policy-Making

Ultimately, the purpose of drawing sufficiency thresholds is to help make policies that fairly allocate benefits and burdens. That does not mean that every discussion of sufficiency must have direct policy implications; but it is reasonable to ask, as two anonymous reviewers have done, how what I have said could be implemented in policy-making. I will not develop a full view of climate policy, or broader distributive justice. But I will briefly address some broad issues with my discussion.

The first issue concerns the measurability of attitudes. Mental states seem a poor basis for policy because they are inaccessible to others. Here, I reiterate the point made above, that as well as self-reports we can depend on other evidence and reliable generalizations about what humans find tolerable and contenting. This evidence is deeply imperfect, and so there are policy decisions to be made about how much we are to trust each type, and how to deal with individuals who may deviate from our otherwise reliable generalizations.

There is clear risk here of making mistakes and giving priority to the wrong individuals in some cases. But the same might be true of many other potential markers of justice. For instance, we judge whether someone is able to live autonomously not by some form of direct access, but through indirect evidence. The key point is that we do not do better with respect to justice by pretending that subjective experience does not exist, or that it is not relevant. To focus exclusively on objective measurements at the theoretical level is to risk confusing an epistemic question with an ethical or political one.

A second issue concerns the relationship between the criteria that I have proposed and the criteria proposed by other sufficientarians, such as autonomy. After all, I have not denied that such further criteria are important for those who can achieve them. How should the criteria set by these separate ideas interact, and what should we do if they clash?

I assume that the state may have a role in protecting both capacities such as those which constitute autonomy, and a level of subjective well-being. Thus, for those who have the relevant capacities, subjective and objective factors set separate criteria for the achievement of sufficiency. As I noted in Section 6, this is an endorsement of ‘horizontal pluralism’. It means, for instance, that someone who is autonomous but finds their situation intolerable has not achieved sufficiency; but nor is someone who finds their situation tolerable but is lacking in autonomy.

But I acknowledge that this may sometimes raise difficult choices. For instance, the state might be forced to choose between protecting subjective tolerability or autonomy (either in the same person, or different people); in other words, we might face a choice between different metrics of sufficiency. And I must admit that I do not have a good answer here as to how to make this decision. But I hope that my argument helps us to see that such a trade-off *is difficult* in a way that an exclusive focus on autonomy would obscure.^4^

## 8. Conclusion

I have argued that we need a theory of sufficiency that pays attention to how people’s lives feel to them from the inside, at the very least to complement measures such as autonomy or freedom. This is because a sole focus on these latter measures excludes some individuals, including those with significant cognitive disabilities. I suggested that while some sufficientarians (notably Frankfurt and Huseby) have allowed for such subjective assessments in grounding a higher threshold, we should also consider them in the form of tolerability in grounding a lower threshold.

Problems remain. A clear worry about sufficientarianism is that it reinforces existing, unjust inequalities by insisting that inequality *per se* does not matter. The view I have outlined exacerbates this worry, since the more powerful and privileged will be accustomed to a much higher quality of life than those who are victims of oppression or injustice. I have offered some considerations that mitigate but do not remove this concern. I suspect that the only way to eliminate it completely is to abandon any concern for our subjective assessments and focus solely on objective goods or capacities as relevant to justice. For reasons outlined in this paper, I do not regard this as a satisfying alternative. But where all the alternatives are unsatisfying, we may have to pick the best of a bad bunch of options. Thus, I take myself only to have outlined an alternative way of thinking about sufficiency, not to have made a comprehensive case for its adoption.

## Notes

1. One might argue that, at a practical level, our assumption that an individual *cannot* achieve or value a particular capacity is often distorted by stereotyping and bias (Nussbaum, 2006; Wong, 2010). But while I agree that individuals’ capacities should not be written off, there are costs (both to the individuals themselves, and to others) in seeking to enable someone to realize capacities they do not have. And it seems to me compatible with resisting ableist bias to believe that there may come a point for some individuals where it is better to reconceive what a good life looks like for them.

2. Hassoun (2021a, 2021b) uses the idea of contentment as a negative test for minimally good lives; if you would not be content with another person’s life, you should not regard it as minimally good. I assume this is a heuristic rather than a substantive threshold, and so I concentrate on Frankfurt and Huseby.

3. Frankfurt (1987) explicitly rejects appeals to tolerability (p. 38). But he seems explicitly concerned with a threshold where people’s claims cease.

4. Although I have focused on individuals for whom sufficiency can only depend on subjective facts, there may also be individuals for whom the opposite is true. For instance, an anonymous reviewer raises the issue of individuals who are unconscious and entirely lacking in subjective experience. It is worth noting, though, that such an individual will also lack the key *objective* factors on which sufficientarians have focused. Thus, although our immediate concern may be with objective criteria such as bare survival, I assume that treatment would also be decided in reference to longer-term objective and subjective goals.
